# Hardness and Roughness of Glass/Epoxy Composite Laminates Subjected to Different Hostile Solutions: A Comparative Study

**DOI:** 10.3390/polym17070993

**Published:** 2025-04-07

**Authors:** Ana Martins Amaro, M. F. Paulino, Maria Augusta Neto, Paulo N. B. Reis

**Affiliations:** University of Coimbra, CEMMPRE-ARISE, Department of Mechanical Engineering, 3030-788 Coimbra, Portugal; maria.paulino@dem.uc.pt (M.F.P.); augusta.neto@dem.uc (M.A.N.)

**Keywords:** glass/epoxy composites, hostile environments, hardness, roughness

## Abstract

This work aims to compare the hardness (H) and roughness (R_a_) of glass/epoxy composites after being exposed to various hostile environments, which is possible because the constituents are always the same. Considering the stacking sequence [45_2_, 90_2_, −45_2_, 0_2_]_s_, the hardness increases for all solutions up to a certain exposure time, from which it decreases for longer immersion times. For the same stacking sequence, roughness had its highest increase (around 44.5%) for the alkaline solution after 36 days of immersion, while the highest decrease (around 25%) occurred for all mortars after 30 days of exposure. For the stacking sequence [0_2_, 90_2_]_2s_, the hardness varied in the opposite direction for acidic and alkaline solutions, observing a direct increase in H with immersion time. However, for samples immersed in oil, hardness decreased as a function of immersion time. In terms of roughness, there was a linear increase with immersion time for all samples, which increased linearly. Therefore, it can be concluded that the stacking sequence has a significant influence on hardness and roughness. Furthermore, knowledge of the variation in hardness and roughness is very important because it can be associated with the structural response of a composite exposed to hostile environments.

## 1. Introduction

Composite materials are increasingly replacing traditional metallic materials in various industrial sectors and this evidence is even noted in components for applications in highly corrosive environments. In this case, the interest in glass-reinforced plastic (FRP) components as an alternative to stainless steel or coated steel ones is becoming common due to the weight reduction they provide, high specific strength and stiffness, competitive cost, good static and dynamic properties, good corrosion resistance, and simplified manufacturing [1,2,3,4,5]. Therefore, composites are very advantageous for different industrial sectors due to these advantages [6,7,8,9,10,11,12].

Based on this enormous interest, it is not surprising that there are several studies available in the literature aimed at understanding degradation phenomena and their effects on mechanical properties to establishing reliable design criteria. In terms of stress corrosion cracking, for example, literature is abundant [13,14,15,16,17,18], and it is a phenomenon that results from the combined action of load and corrosive environments. In this case, sharp cracks appear and propagate subsequently through the material with the consequent weakening/collapse of the reinforcement elements due to the chemical attack that they undergo through the crack tip. According to Bazli et al. [19], an ion exchange occurs when solutions penetrate through voids, cracks, or the fiber/matrix interface affecting the matrix (chain scission) and the fiber/matrix interfacial strength with consequent reduction in mechanical properties. Therefore, it is well recognized that adverse conditions affect the mechanical performance of composite materials throughout their service life [20].

In this context, according to Mahmoud and Tantawi [21], the bending strength, hardness, and Charpy impact strength depend significantly on the type of acid and the immersion time. Regarding HCl exposure, bending strength decreases by about 10% after 30 days of immersion, hardness drops by about 15% after 90 days of exposure, while Charpy impact strength decreases by about 5% in the first 60 days of immersion and by 10% between 60 and 90 days. For Mahmoud and Tantawi [21] the exposure time is a determining parameter in mechanical performance that must be considered in design considerations. The impact of exposure to water and alkaline solution on strength was evaluated by Won et al. [22], who corroborated the findings of previous authors by observing that the tensile strength of GFRP was significantly affected by the exposure time. Banna et al. [23] compared the exposure to two acidic solutions of a polyester resin and a bisphenol A epoxy vinyl ester resin for different times and temperatures, observing that the polyester resin was the most affected by the temperature and exposure time, evidence reported by the higher increase in surface roughness, cracks, and diffusion of sulfur into the cracks. The hardness of both resins increased after two weeks and decreased further after 4 weeks, but to higher values than those observed for the unexposed resins. When exposed to alkaline environments, the vinylester and epoxy resins exhibited the lowest degradation rate, while the polyester composites showed the highest degradation rate, according to Benmokrane et al. [24]. Some authors concluded that increasing the pH of the alkaline solution promotes a decrease in tensile strength and modulus, while an increase was observed for acidic solutions [25,26]. Pai et al. [27] studied the effects of increasing the sulfuric acid concentration and the sequential layup and observed that composites with chopped strand mat showed a greater increase in mass gain than those with woven roving mat as the skin layers due to the hydrolytic dissolution of the matrix in contact with the acid [25,27]. Sindhu et al. [28] compared different solvents and concluded that tensile properties increase in acidic environments. Kawada and Srivastava [18] observed that stress corrosion cracking of GRP occurs due to the combined action of loading and exposure to a corrosive environment. Sharp cracks begin and spread through the material as a direct consequence of the weakening of the glass fibers by the acid. Fiber strength is drastically reduced due to acid diffusion and chemical attack of the fiber surface at the crack tip, which causes a highly flat fracture with a greatly reduced failure stress. The degradation of fiber matrix interfaces cannot be excluded because the degradation of the resin/fiber interface, due to physical and chemical interactions, also explains the lower mechanical properties observed in the composites [29,30,31,32].

Amaro et al. [33] analyzed the exposure of a glass fiber/epoxy composite to hydrochloric acid (HCl) and sodium hydroxide (NaOH) solutions, and the ultramicroindentation results showed a decrease in the matrix mechanical properties, as well as an increase in roughness with exposure time, but with more expressive values for samples immersed into NaOH. Furthermore, the alkaline solution proved to be more aggressive than the acidic solution, promoting lower bending properties and impact strength. In another very similar study [34], the authors analyzed the effect of exposure to hydrochloric acid (HCl) and sulfuric acid (H_2_SO_4_) solutions and obtained similar results to the previous study [33], but with hydrochloric acid responsible, in this case, for the lower bending properties and impact strength. Mortas et al. [35] studied the low-velocity impact response of carbon/epoxy and Kevlar/epoxy laminates after immersion into hydrochloric acid (HCl) and sodium hydroxide (NaOH) at various concentrations and temperatures. These authors concluded that, regardless of the laminates and solutions, the impact response is strongly affected by the concentration and temperature of the solutions, but with greater severity for the alkaline solution. Finally, the effect of these solutions was extended to tests at high strain rates, using a Split Hopkinson Pressure Bar (SHPB), Advance Instrument Inc., Norwood, MA, USA, and it was concluded that, regardless of the solution, stress decreases, and strain increases as well as strain rate [36]. Hota et al. [37] corroborate the results of these authors, noting that higher pH and temperatures lead to ILSS losses of up to 30% after 80 to 100 days of aging. The low-velocity impact response of Kevlar/epoxy composites after immersion into diesel, H_2_SO_4_, HCl, NaOH, destined water, and seawater was analyzed by Reis et al. [38], and the authors found that impact strength is substantially affected by exposure to these solutions, but with especial relevance to seawater and NaOH. Subsequently, the residual fatigue life was shown to be essentially governed by the severity of the damage introduced.

After exposing carbon fiber laminates with a neat epoxy matrix and another filled with cork powder to solutions including diesel, H_2_SO_4_, HCl, NaOH, distilled water, seawater, and seawater at 60 °C, Silva et al. [39] investigated their response to low-velocity impact and observed that all solutions affect the impact strength, but the severity of each depends largely on the immersion time. Moreover, cork powder brought benefits in terms of perforation threshold and impact strength. Before and after immersion into HCl and NaOH solutions, Kamal and Kadhim [40] investigated the tensile, impact, and hardness response of ceramic-reinforced polyester hybrid composites containing nano-silica particles. They found that all properties are affected by both solutions, but the alkaline solution was more aggressive than the acidic one, and the exposure time controlled the degradation rate. Nagendiran et al. [41] observed that, compared to neat resin, an epoxy matrix filled with oil fly ash has greater chemical resistance to acidic and alkaline solutions and, consequently, the mechanical properties are less affected. The effects of various solutions on glass fiber-reinforced epoxy composites were analyzed by Feng et al. [42], and they found a decrease in hardness, bending strength, and modulus, which increased with increasing concentrations. Moreover, Ancas et al. [43,44] found that this decrease is proportional to the deviation from neutral pH. Finally, at stress relaxation level, the literature reports that, regardless of the corrosive solution, stress decreases over time but is strongly dependent on the exposure time, temperature, and solution concentration [45,46].

From the above-mentioned state of the art, it is possible to conclude that the exposure of composite materials to various corrosive environments affects their mechanical performance and, in fact, they are increasingly in contact, for example, with corrosive solutions (acidic and alkaline), oils, mortar solutions, etc. In this context, the impact on structural integrity may even be related to the hardness and roughness observed after immersion in different hostile environments and, thus, their mechanical response can be expected. Moreover, in terms of roughness, for example, R_a_ (arithmetic mean roughness) is the most used parameter and represents the average of the variations in height of the surface in relation to the mean line. Therefore, this parameter can provide early evidence of microcrack formation, which is very useful for predicting the mechanical response of the composites. In fact, to the authors’ knowledge, no one has analyzed in detail the effect of harsh environments on the hardness and roughness of composite laminates, so this study is very important to challenge this cause/effect relationship in the scientific community. For this purpose, laminated composites produced with epoxy matrix and glass fibers subjected to different solutions will be considered to establish their response to different aggressive environments.

## 2. Materials and Methods

In this study, two different stacking sequences, both with 16 plies, were used: [45_2_, 90_2_, −45_2_, 0_2_]_s_ and [0_2_, 90_2_]_2s_. The laminates were prepared with glass fiber Prepreg TEXIPREG^®^ ET443 (EE190 ET443 Glass Fabric PREPREG from SEAL, Legnano, Italy), using the autoclave/vacuum bag molding process, in agreement with the manufacturer’s recommendations. For this purpose, the processing setup involved several steps: Initially, the prepregs are placed in a hermetic bag and a vacuum of 0.05 MPa is applied; heat up to 125 °C at a 3–5 °C/min rate and when the temperature is reached a pressure of 0.5 MPa is applied; maintain pressure and temperature for 60 min, followed by cooling to room temperature at constant pressure; and finally, remove the plate from the bag. Using this methodology, the volume fraction of E-glass fiber in these composites was 44.5%. The immersion was performed in an alkaline solution (sodium hydroxide (NaOH)), and into two acidic solutions (hydrochloric acid (HCl) and sulphuric acid (H_2_SO_4_)). The three solutions presented a concentration of 10% in weight (wt.%) and pH level of 13.0, 1.5, and 1.5, respectively, for NaOH, HCl, and H_2_SO_4_. Three mortars solutions were considered: cement mortar, which was produced with sand, cement, water, and a plasticizer, using an electric mixer; cement mortar with water, where some specimens were immersed into water, just after hardening; geopolymers mortar, which has sodium hydroxide, metakaolin powder, and sodium silicate in its composition. In the case of immersion into mortar, the boxes built for immersing the specimens were then wrapped in plastic film to reduce, or prevent, water exchange with the environment, to reduce shrinkage caused by dehydration and thus improve the curing process. Also, two different oils were considered, one less aggressive than the other, 15W40 (a universal multi-grade engine oil) and DOT4 (an extra high-performance hydraulic brake fluid), respectively. All the samples were completely submerged, considering different exposure times.

According to the BS EN ISO 62:1999 standard [47], the following procedure was applied to determine solution absorption: the samples were placed in an oven at 40 °C for 6 h, then cooled and weighed to determine the dry weight (DW). Subsequently, a set of samples was immersed in the respective hostile solutions and periodically weighed to determine the current wet weight (CWW). The solutions absorption, in weight percentage (W%), was obtained according to Equation (1):

(1) $$W\%=\frac{CWW-DW}{DW}\times 100\%$$

To evaluate the influence of these different hostile environments on the hardness and surface roughness of the laminates, experimental tests were carried out using specific equipment. In this case, after exposure to corrosive environments, hardness was assessed by ultramicroindentation using a Fisherscope H100 device (Helmut Fischer GmbH, Sindelfingen, Germany) and a load of 500 mN for 40 s, with an average of 11 passes being made on each specimen along the length and 4 along the width. It should be noted that to maximize the reliability of the established comparisons, the hardness values were corrected for the geometric imperfections of the Vickers indenter, the thermal drift of the equipment, and the uncertainty in the zero position as recommended by Antunes et al. [48]. Furthermore, hardness was obtained by dividing the maximum load applied during the indentation test by the indentation contact area immediately prior to unloading. The roughness profiles were obtained using a Mitutoyo model SJ-500 (Mitutoyo, Kawasaki, Japan). In order to assess the influence of hostile solutions, hardness and roughness were also obtained on control samples using the same techniques and equipment. In all conditions/samples at least 10 valid analyses/tests were performed.

In this context, it possible to compare the control samples with the samples immersed in the hostile environment and analyze the influence on the composite’s response. Table 1 summarizes all hostile conditions used in the present study, where the notation for the stacking sequence adopted square brackets, in which distinct layers or groups of layers are separated by a comma (,), the subscript number (n) designates the number of repeating groups or layers, and the symmetrical laminates are designated by the subscript S in square brackets. Finally, the stacking sequences [45_2_, 90_2_, −45_2_, 0_2_]_s_ and [0_2_, 90_2_]_2S_ are characterized by having the same number of layers, and consequently the same number of interfaces, but the first one ensures that the laminate has almost equal stiffness and strength in both the longitudinal, transverse, and shear directions. Therefore, this quasi-isotropic laminate design provides high strength and stiffness while maintaining some degree of flexibility, making it ideal for applications where strength and lightness are essential for aerospace, automotive, and sporting equipment. Therefore, the results obtained from this experimental campaign will be presented in 4 subsections, in which the first evaluates the mass variation and the others aim to make a comparative analysis between the acid/alkaline solutions and the mortar solutions, between the acid/alkaline solutions and the different oils, and, finally, evaluate the effect of the stacking sequence.

## 3. Results

### 3.1. Weight Gain

Table 2 summarizes the effect of the different hostile solutions (identified in Table 1) and exposure time on weight gain, and it is possible to observe a significant influence in terms of variation in the mass of the specimens, depending on the solution in which they were immersed. The most aggressive solutions in terms of mass influence are acidic and alkaline solutions, which promote the greater increase, identified with a blue circle. In the case of mortar solutions, the increase in mass is not significant, with values of less than 1%. A slight tendency for weight gain can be observed with the 15W40 solution; however, an opposite trend is noted for the DOT4 solution, labeled with a green circle [49]. Specifically, after 45 days of exposure, a decrease of around 2.16% is observed compared to the dry samples. Furthermore, analyzing the mass increase for the stacking sequence [45_2_, 90_2_, −45_2_, 0_2_]_s_ immersed into HCl for 36 days and the mass increase for the same solution for 90 days for the stacking sequence [0_2_, 90_2_]_2s_, it can be observed that the mass increase is about 21% higher for the first stacking sequence, even though it is immersed for a shorter time. The same trend is observed for the alkaline solution, although with a greater difference (in this case greater than 200%). When the acid solution comes into contact with the glass fibers, an ion exchange occurs on the surface of the glass and the hydrogen ions found in the acidic solution. When the solution is alkaline, the chemical reaction involves a breakdown of the network by hydroxide ions (OH), which eventually promotes dissolution of the E-glass [50]. This creation of microcracks leads to the appearance of holes, which increases with the exposure time. The solution will be introduced into these voids, promoting an increase in mass until reaching a saturation point, at which point the mass may decrease due to the reduction in the density of the external part of the fiber and shortening of the fiber length. Finally, the variation observed in the increase in mass between samples immersed in aqueous solutions (acidic and alkaline) and those immersed in colloidal suspensions (cement and geopolymer), for the same fiber orientation, can be explained by the difference in viscosity between the two types of adverse environments, which influences the capacity for absorption and adsorption.

### 3.2. Comparison Between Acid/Alkaline Solutions and Mortars

To analyze the effects of acid/alkaline solutions and mortars on hardness and roughness, samples with the stacking sequence [45_2_, 90_2_, −45_2_, 0_2_]_s_ were used. This comparison is due to the fact that the mortar solutions are alkaline-based (i.e., contain NaOH). Furthermore, the difference in immersion times is justified because no significant effect of the mortars was observed for shorter times. According to Banna et al. [23], the microhardness tests were found to be one of the most sensitive to the exposure condition. For acidic and alkaline solutions, the samples were immersed for 12, 24, and 36 days, whereas for mortar solutions, the exposure times were 30, 60, and 90 days. Table 3 summarizes the results in terms of average and standard deviation (±) of the effect of corrosive environments on the hardness (H), indentation modulus (E_R_), and Young’s modulus (E) of the matrix. From this table, it is possible to observe that the most significant increase in hardness, considering the acidic and alkaline solutions, occurs for samples exposed to H_2_SO_4_ for 12 days. In fact, for both solutions, the hardness increases up to a maximum value from which it decreases with the exposure time. In the present study, the highest value occurs for 12 days of immersion, a trend that is in line with the studies of Banna et al. [23]. This can be justified due to changes in the chemical structure that occur in the epoxy matrix [51,52,53,54]. It is also possible to observe that the alkaline solution is not so sensitive to immersion time, promoting lower hardness variation values than those observed for the specimens immersed in sulfuric acid, which is in accordance with what was found in [42]. In this context, the increase in hardness and modulus after immersion can be justified by the increase in the number of secondary bonds observed, despite the structure not being reticulated. Furthermore, after immersion, the matrix degrades so that the fibers are more exposed and, therefore, an increase in hardness occurs.

In the case of immersion into mortar solutions, the variation in hardness shows the greatest increase in values, particularly in the case of cement with water. However, the greatest increase is observed in the case of immersion in cement for 30 days. According to Amaro et al. [55], considering the composites immersed into mortar solutions, the main damage mechanism observed is the matrix cracking and rupture of the fibers. Therefore, as the matrix does not protect the fibers, when the hardness is assessed, the fibers are more exposed, so that during indentation it is not the matrix that responds to the indenter, but the fiber, which promotes an increase in the hardness of the composite. The increase in hardness for samples immersed in acidic and alkaline solutions can be attributed to the formation of secondary bonds, such as hydrogen ones [21]. On the other hand, comparing the values of hardness for the alkaline solution with those of the geopolymer, it can be noticed that they are in the same order of magnitude. This fact can be justified due to the geopolymer containing sodium hydroxide in its composition. Immersion times are significantly different when comparing acid and alkaline solutions with mortars, since mortars, being colloidal suspensions, need more time for ions to diffuse into the matrix, so that absorption and adsorption can be observed.

**Figure 1:**
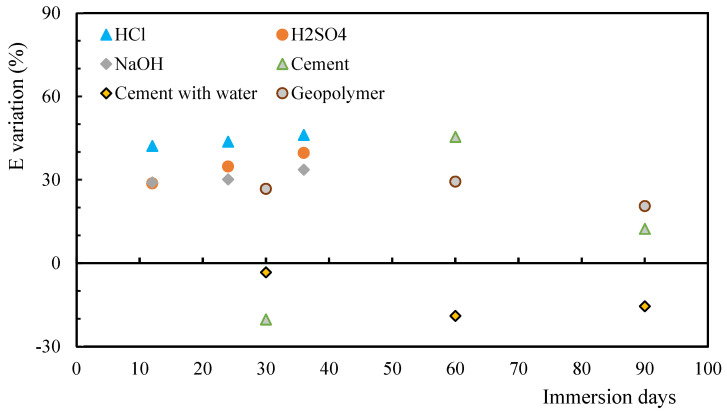
Matrix of Young’s modulus (E) for alkaline/acid solutions and mortars.

In terms of variation in Young’s modulus of the matrix (*E*), it is possible to observe from Figure 1 that in the case of cement with water, there was an increase in the *E* value for all immersion times. For immersion in cement for 30 days, an increase in the *E* value is also detected. For all the other degradation conditions, a decrease in the *E* values were found. It is similarly possible to note that the decrease observed for the geopolymer is of the same order of magnitude as that of the alkaline solution, in line with what was justified for the variation in terms of hardness. Therefore, the distribution and adhesion of reinforcements to the matrix can affect the hardness and modulus differently. If the reinforcements are well distributed and strongly joined, the hardness may increase more than the modulus, or in other situations the opposite may occur. Although the composites were inspected prior to testing to verify that there were no manufacturing defects, depending on the chemical reactions observed between the composite and the solutions, there may be different structural responses to different conditions. Moreover, while hardness and modulus are generally correlated, the complexity of composites can lead to deviations due to microstructural or mechanical factors.

**Figure 2:**
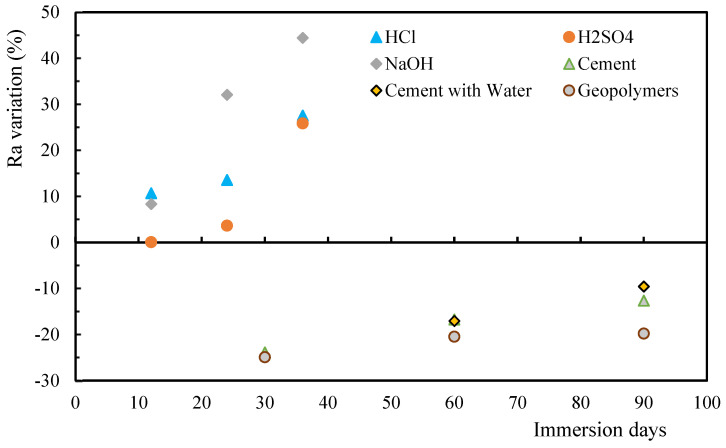
Roughness (R_a_) values for alkaline/acid solutions and mortars.

The presence of microcracks was analyzed based on the roughness profiles, where Figure 2 highlights the effect of the solutions on the surface topology, as well as the effect of the exposure time. Regarding the arithmetic mean roughness (*R_a_*) of the mortar solutions, Figure 2 presents a negative variation, while for the acid and alkaline solutions it is positive, which means that there is an increase in the case of acid and alkaline solutions and a decrease for the mortars. For *R_a_*, the largest increase (around 44.5%) was observed for NaOH and 36 days of immersion, while the largest decrease (around 25%) occurred for all mortars and after 30 days of exposure. The mortars always suffered a decrease in roughness value and, as the immersion time increased, this decrease was reduced. However, the geopolymers showed the greatest decrease for all immersion times when compared to the other two mortar solutions. On the other hand, for acidic and alkaline solutions the roughness always increased, with the immersion time, but with increasing immersion time this increase became smaller. Therefore, it is possible to conclude that the type of environment significantly affects the roughness of the material, which will promote changes in the structural integrity of the composite.

In the case of the mortars, the main explanation for this significant initial reduction in roughness may be related to the solutions filling the porosities in the specimen. This could make the surface of the specimen more uniform, attenuating possible microcracks that initially existed. The similarity in terms of roughness when comparing the specimens immersed in cement and cement with water can be explained by the fact that these solutions are made up of the same constituents, differing only in the medium in which they are cured. Therefore, due to the similarity in evolution, it can be concluded that immersion in water does not directly affect the roughness of the specimens but only delays the curing of the solution and promotes the transport of particles into the pores of the specimen.

### 3.3. Comparison Between Acid/Alkaline Solutions and Oils

Considering the stacking sequence of [0_2_, 90_2_]_2s_, the acidic (HCl) and alkaline (NaOH) solutions were studied for 15, 30, 60, and 90 days, while the oils were studied for 15 and 45 days. This comparative analysis is justified by the different chemical reactions that occur between the composite and the different solutions, while in terms of exposure time it was found that the samples immersed into oil are affected for shorter times than the others. In this case, the hardness assessment was also carried out on at least 11 indentations along a line in the longitudinal dimension of the specimen and at least four indentations in the transverse dimension.

**Figure 3:**
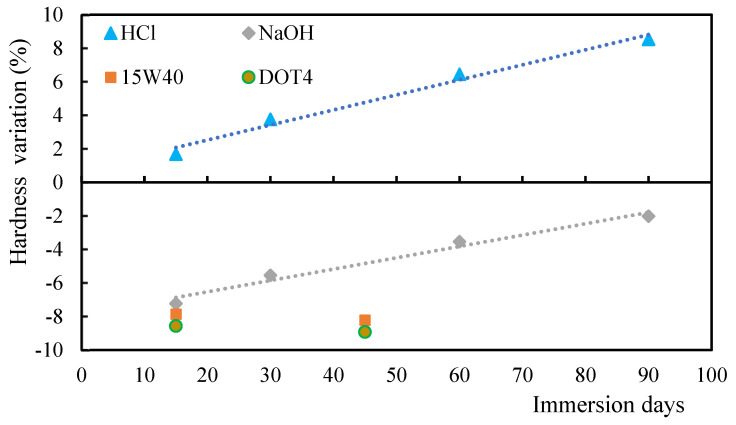
Hardness (H) variation for alkaline/acid solutions and oils.

It is possible to observe from Figure 3 that the hardness increases with the immersion time in the case of the acid solution, while in the case of the alkaline solution there is a decrease in the hardness values, both when compared to the control samples. In this case, the variation in the values of hardness for both acid and alkaline solutions is almost linear with immersion time. Therefore, the decrease in hardness when the samples are immersed into oils can be explained by the fact that oils have a plasticizing effect and do not resist indentation. As the immersion time increases, the hardness value decreases in line with the studies of Banna et al. of a significant number of cracks [49]. In fact, immersion in acidic and alkaline solutions increases the interaction of molecules within the matrix itself, creating new hydrogen bonds and, although covalent bonds are not formed, an increase in secondary bonds is observed, which impacts the properties under study. On the other hand, oils are chains of fatty acids with distance between the macromolecules that constitute them, but no breaking of covalent bonds is observed. Furthermore, as reported above, because they have a plasticizing effect, the hardness value decreases.

**Figure 4:**
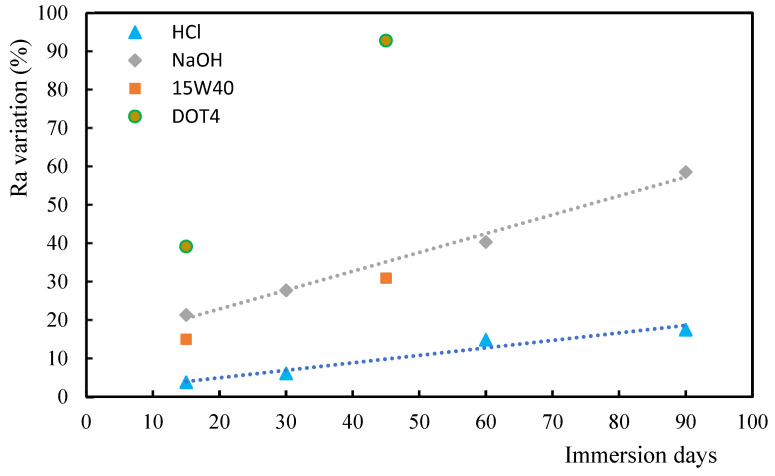
Roughness (R_a_) variation for alkaline/acid solutions and oils.

From Figure 4, which presents the roughness comparison, it is noticed that the greatest increase was observed for samples immersed into DOT4 oil (an aggressive oil) for 45 days. On the other hand, for 15 days of immersion, the highest increase in the *R_a_* value is observed for DOT4, around 39%, followed by NaOH (21%), 15W40 (15%), and HCl (4%). A linear trend is observed in the case of acid and alkaline solutions, with roughness increasing as a function of immersion time, like that observed for hardness. Exposure to oils increases roughness due to the appearance of multiple cracks in terms of matrix and matrix/fiber interface. However, due to the more aggressive effect of DOT4, there is also a loss of adhesion between fibers and matrix in samples immersed into DOT4 [49]. This effect is not as significant in samples immersed in 15W40, because this oil has a higher viscosity than DOT4, which is justified by the fact that 15W40 contains hydrocarbon-based compounds. The highest value of the increase in *R_a_* is evaluated for the samples immersed into DOT4 for 45 days, with a value of around 93%.

### 3.4. Influence of Stacking Sequence

**Figure 5:**
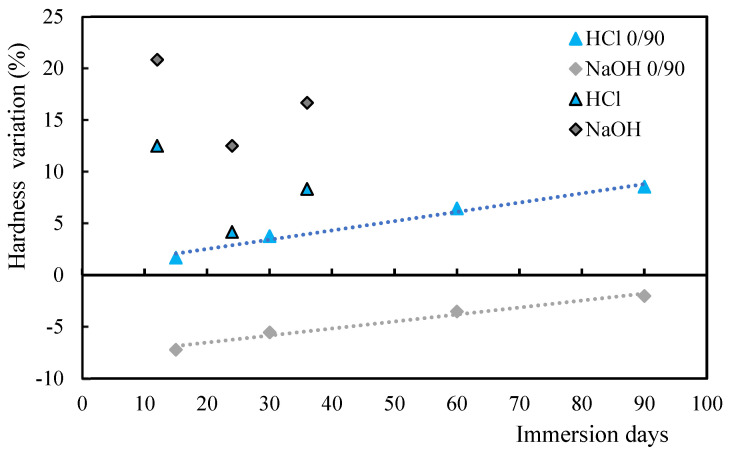
Hardness variation (H) for samples with different stacking sequences. HCl 0/90 and NaOH 0/90 is for samples with stacking sequence of [0_2_, 90_2_]_2s_, HCl and NaOH for samples with stacking sequence of [45_2_, 90_2_, −45_2_, 0_2_]_s_.

**Figure 6:**
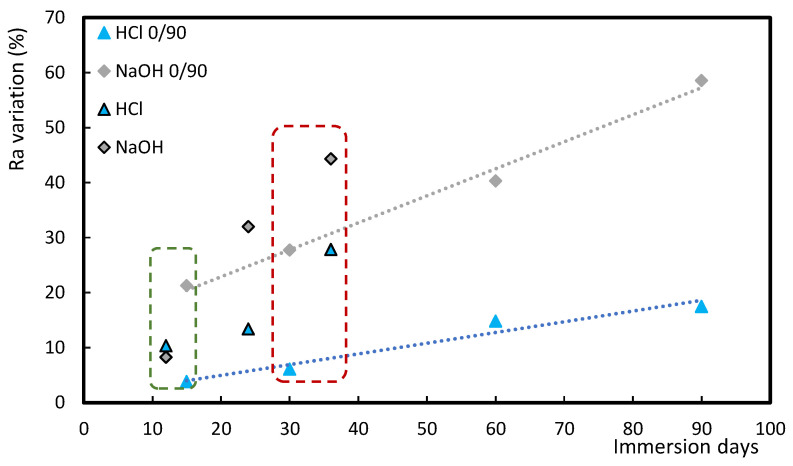
Roughness (R_a_) variation for samples with different stacking sequences. HCl 0/90 and NaOH 0/90 is for samples with stacking sequence of [0_2_, 90_2_]_2s_, HCl and NaOH for samples with stacking sequence of [45_2_, 90_2_, −45_2_, 0_2_]_s_.

Samples with the stacking sequence of [45_2_, 90_2_, −45_2_, 0_2_]_s_ and [0_2_, 90_2_]_2s_ and immersion times of 12, 24, and 36 days, and 15, 30, 60, and 90 days, respectively, were used. It is worth noting that in this study only HCl and NaOH solutions were considered because, as shown in Table 3, the former affected the parameters under study more than the H_2_SO_4_ solution. In this context, the analysis of both acids (HCl and H_2_SO_4_) would be redundant and would lead to the same conclusion. Furthermore, due to the number of interfaces between layers with different orientations being different (although both have 16 layers), this may lead to a different degradation rate and therefore different immersion times were used. Therefore, according to Figure 5, the stacking sequence has a significant influence on the hardness of the samples. It is noticed that the greatest increase in hardness value is observed in the case of the specimens immersed into NaOH for 12 days and for the stacking sequence of [45_2_, 90_2_, −45_2_, 0_2_]_s_, while for the same solution and [0_2_, 90_2_]_2s_ stacking sequence there is observed a decrease in hardness value for 15 days of immersion. On the other hand, for the [0_2_, 90_2_]_2s_ stacking sequence and for both solutions a linear trend is observed for the variation in hardness as a function of immersion time. However, for the stacking sequence [45_2_, 90_2_, −45_2_, 0_2_]_s_ this trend is not observed, but an increase followed by a decrease and then again an increase with increasing time is observed. Moreover, Figure 6 shows that for lower immersion times, the greatest increase in roughness is observed for samples immersed in NaOH for 15 days, with the stacking sequence of [0_2_, 90_2_]_2s_. However, as the immersion time increases, a reversal is observed, with the samples with the sequence of [45_2_, 90_2_, −45_2_, 0_2_]_s_, also immersed into NaOH, showing the greatest increase in the *R_a_* value. It can also be noticed that the alkaline solution promotes the greatest increase in *R_a_* values for all immersion times and for both stacking sequences. In agreement with what was observed for hardness, for roughness it was also not possible to find a linear variation with increasing *R_a_* values and immersion time for the stacking sequence of [45_2_, 90_2_, −45_2_, 0_2_]_s_.

## 4. Conclusions

It was observed that in the case of alkaline and acidic solutions, for the stacking sequence of [45_2_, 90_2_, −45_2_, 0_2_]_s_, the greatest increase in hardness was obtained for the shortest exposure time, despite an increase in hardness being observed for all exposure times under study. The highest value is detected for samples immersed in sulfuric acid for 12 days, with a value of 45.8%. Considering cement for 30 days of immersion the great increase in hardness was around 79%. The increase in hardness of the geopolymer is of the same order of magnitude as that observed for the alkaline solution, even though different times are being compared. However, as explained above, colloidal solutions require more time for the ions to diffuse into the matrix, so it was necessary to consider longer immersion times for the mortars.

In terms of roughness, the same trend is observed, or in other words, there is an increase in roughness for samples immersed in acid and alkaline solutions, and a decrease for samples immersed in mortars, the greatest increase being evaluated for samples immersed during 36 days into alkaline solution (NaOH) of around 84%. Comparing the influence of acid and alkaline solutions with the influence of oils, for the same stacking sequence, [0_2_, 90_2_]_2s_, the aggressive oil (DOT4) promotes the greatest increase in roughness of around 127%. Even after 90 days of immersion into acidic and alkaline solutions, there is no approximation between the roughness increase values of the specimens immersed in these solutions and those immersed in DOT4.

The stacking sequence has a great influence on the variation in hardness and roughness. The samples with orientation [45_2_, 90_2_, −45_2_, 0_2_]_s_ show higher values for the increase in hardness than the samples with the stacking sequence of [0_2_, 90_2_]_2s_. On the other hand, for the samples with [45_2_, 90_2_, −45_2_, 0_2_]_s_ there is no linear variation in the hardness as a function of time, which can be noticed for samples with the [0_2_, 90_2_]_2s_ lay-up. The greatest change in behavior is observed for samples immersed into NaOH where, in the case of [0_2_, 90_2_]_2s_, there is a linear decrease in its value as a function of time, while for [45_2_, 90_2_, −45_2_, 0_2_]_s_ and 12 days of immersion there is the greatest increase in hardness value.

For roughness, as with hardness, there is a linear trend of increasing roughness with immersion time for samples with a stacking sequence of [0_2_, 90_2_]_2s_ which is not observed for the other stacking sequence under study, although there is also an increase in roughness with immersion time. Samples with [0_2_, 90_2_]_2s_ show significantly less roughness increase than those with the other stacking sequence, even for shorter immersion times.

There are many studies that have evaluated the mechanical properties of composites, but there is no comparison between the response of composites with the same matrix and the same fiber, the same number of plies, with different configurations, immersed into various hostile environments. Therefore, these conclusions can help researchers in making decisions about the type of composite to be used, regarding its application, because these variations in hardness and roughness will influence the structural response of the composite when subjected to different loading types. Furthermore, this study also aims to promote discussion on a better application of these materials in chemical processing plants (pipes, storage tanks, and vessels), the oil and gas industry (to handle corrosive fluids and gases for which traditional metals are not recommended), fuel systems that must withstand chemical exposure without losing structural integrity in both water treatment and desalination, and in the automotive sector.

Finally, although roughness has been used to study the possible existence of microcracks caused by hostile environments in the matrix, and consequent impact on the fiber/matrix interface, it is not clear that this parameter alone can reliably and reasonably characterize them, for example, at the level of microcrack length and area. In this context, it is suggested that more appropriate methodologies for this purpose be used in future work. Furthermore, a comparative study is suggested that can relate roughness and hardness with the mechanical performance of composites for different loading types.

## Figures and Tables

**Table 1:** Hostile conditions and properties evaluated.

Stacking Sequence	Hostile Solution	Immersion Time (Days)	Properties
[45_2_, 90_2_, −45_2_, 0_2_]_s_	HCl	12/24/36	Hardness/roughness
	H_2_SO_4_		
	NaOH		
[45_2_, 90_2_, −45_2_, 0_2_]_s_	Cement	30/60/90	Hardness/roughness
	Cement with water		
	Geopolymers		
[0_2_, 90_2_]_2s_	HCl	15/30/60/90	Hardness/roughness
	NaOH		
[0_2_, 90_2_]_2s_	15W40	15/45	Hardness/roughness
	DOT4		

**Table 2:** Effect of the solutions and exposure time on the weight gain.

Stacking Sequence	Hostile Solution	Immersion Time (Days)	Weight Gain (%)	Std. Dev. (%)
[45_2_, 90_2_, −45_2_, 0_2_]_s_	HCl	12	1.83	0.07
		24	4.15	0.08
		36	**4.74**	0.10
[45_2_, 90_2_, −45_2_, 0_2_]_s_	H_2_SO_4_	12	2.78	0.03
		24	3.68	0.05
		36	**5.24**	0.07
[45_2_, 90_2_, −45_2_, 0_2_]_s_	NaOH	12	1.23	0.07
		24	3.26	0.09
		36	**4.17**	0.09
[45_2_, 90_2_, −45_2_, 0_2_]_s_	Cement	30	0.18	0.02
		60	0.13	0.01
		90	0.11	0.03
[45_2_, 90_2_, −45_2_, 0_2_]_s_	Cement with water	30	0.30	0.02
		60	0.55	0.03
		90	0.97	0.03
[45_2_, 90_2_, −45_2_, 0_2_]_s_	Geopolymers	30	0.13	0.02
		60	**0.15**	0.004
		90	0.22	0.003
[0_2_, 90_2_]_2s_	15W40	15	0.21	0.08
		45	0.38	0.12
[0_2_, 90_2_]_2s_	DOT4	15	−0.43	0.14
		45	**−2.16**	0.18
[0_2_, 90_2_]_2s_	HCl	15	0.21	0.001
		30	1.57	0.03
		60	2.19	0.13
		90	3.93	0.05
[0_2_, 90_2_]_2s_	NaOH	15	0.16	0.05
		30	0.64	0.02
		60	1.18	0.17
		90	1.37	0.14

**Table 3:** Effect of hostile solutions on the hardness parameters [33,34,55].

Hostile Solution	Immersion Time (Days)	H (GPa)	E_R_ (MPa)	E (GPa)
Control	-	0.24c0.01	13.12 ± 0.94	11.64 ± 0.91
HCl	12	0.27 ± 0.01	7.60 ± 0.20	6.73 ± 0.15
	24	0.25 ± 0.01	7.43 ± 0.21	6.53 ± 0.14
	36	0.26 ± 0.01	7.10 ± 0.57	6.25 ± 0.48
H_2_SO_4_	12	**0.35 ± 0.09**	9.37 ± 0.75	**8.27 ± 0.62**
	24	0.33 ± 0.02	8.53 ± 0.93	7.57 ± 0.81
	36	0.29 ± 0.03	7.85 ± 0.64	7.00 ± 0.57
NaOH	12	0.29 ± 0.01	9.30 ± 0.44	8.23 ± 0.42
	24	0.27 ± 0.01	9.15 ± 0.67	8.10 ± 0.58
	36	0.28 ± 0.01	8.70 ± 0.64	7.70 ± 0.58
Cement	30	**0.43 ± 0.02**	15.08 ± 0.61	**13.95 ± 0.49**
	60	0.29 ± 0.01	6.89 ± 0.52	6.33 ± 0.23
	90	0.26 ± 0.02	11.09 ± 0.72	10.17 ± 0.42
Cement with water	30	**0.35 ± 0.02**	13.02 ± 0.68	**11.98 ± 0.57**
	60	**0.38 ± 0.02**	15.24 ± 0.75	**13.80 ± 0.61**
	90	**0.37 ± 0.03**	14.50 ± 0.65	**13.40 ± 0.52**
Geopolymers	30	0.29 ± 0.01	10.28 ± 0.56	9.45 ± 0.34
	60	0.28 ± 0.02	8.98 ± 0.43	8.20 ± 0.25
	90	0.25 ± 0.02	10.02 ± 0.62	9.22 ± 0.32

## Data Availability

Provided upon request.
